# Cost analysis between mini-percutaneous nephrolithotomy with and without vacuum-assisted access sheath

**DOI:** 10.1007/s00345-021-03811-5

**Published:** 2021-08-25

**Authors:** Elena Lievore, Stefano Paolo Zanetti, Irene Fulgheri, Matteo Turetti, Carlo Silvani, Carolina Bebi, Francesco Ripa, Gianpaolo Lucignani, Edoardo Pozzi, Lorenzo Rocchini, Elisa De Lorenzis, Giancarlo Albo, Fabrizio Longo, Andrea Salonia, Emanuele Montanari, Luca Boeri

**Affiliations:** 1 Department of Urology, IRCCS Foundation Ca’ Granda, Ospedale Maggiore Policlinico, University of Milan, Via della Commenda 15, 20122 Milan, Italy; 2 Department of Radiology, Foundation IRCCS Ca’ Granda – Ospedale Maggiore Policlinico, Milan, Italy; 3 Division of Experimental Oncology/Unit of Urology, URI, IRCCS Ospedale San Raffaele, University Vita-Salute San Raffaele, Milan, Italy; 4 Department of Clinical Sciences and Community Health, University of Milan, Milan, Italy

**Keywords:** Percutaneous nephrolithotomy, Vacuum-assisted percutaneous nephrolithotomy, Cost analysis, Urolithiasis, Infectious complications

## Abstract

**Purpose:**

To perform a cost analysis between vacuum-assisted percutaneous nephrolithotomy (vmPCNL) and minimally invasive PCNL (MIP) and explore potential predictors of costs associated with the procedures.

**Methods:**

We analyzed data from 225 patients who underwent vmPCNL or MIP at a single tertiary referral academic center between January 2016 and December 2020. We collected patients’ demographics, peri-and postoperative data and detailed expense records. After propensity score matching, 108 (66.7%) vmPCNL and 54 (33.3%) MIP procedures were analyzed. Descriptive statistics assessed differences in clinical and operative parameters. Univariable and multivariable linear regression models tested the association between clinical variables and costs.

**Results:**

Operative time (OT) was shorter for vmPCNL, and the use of additional instruments to complete litholapaxy was more frequent in MIP (all *p* ≤ 0.01). Length of stay (LOS) was longer for MIP patients (*p* = 0.03) and the stone-free (SF) rate was higher after vmPCNL (*p* = 0.04). The overall instrumentation cost was higher for vmPCNL (*p* < 0.001), but total procedural costs were equivalent (*p* = 0.9). However, the overall cost for the hospitalization was higher for MIP than vmPCNL (*p* = 0.01). Univariable linear regression revealed that patient’s comorbidities, OT, any postoperative complication and LOS were associated with hospitalization costs (all *p* < 0.001). Multivariable linear regression analysis revealed that LOS and OT were associated with hospitalization costs (all *p* < 0.001), after accounting for vmPCNL procedure, patients’ comorbidities, and complications.

**Conclusion:**

vmPCNL may represent a valid option due to clinical and economic benefits. Shorter OT, the lower need for disposable equipment and the lower complication rate reduced procedural and hospitalization costs.

**Supplementary Information:**

The online version contains supplementary material available at 10.1007/s00345-021-03811-5.

## Introduction

Percutaneous nephrolithotomy (PCNL) is recommended as the standard procedure for large renal stones [1]. However, the introduction of miniaturized instrumentation has widened the indications of PCNL to a greater range of stone volumes [2]. Miniaturized PCNL was found to be as effective as standard procedure, but with decreased morbidity rates, bleeding, postoperative pain and shorter hospitalization [3, 4]. On the contrary, major drawbacks of miniPCNL are longer operative time (OT), decreased visibility and higher intra-pelvic pressure, which is associated with postoperative infectious complications [5, 6].

Recently, new technologies have been introduced to decrease the limitations of a smaller tract size. During standard PCNL, suction has been used mainly combined with ultrasound and ballistic devices to facilitate litholapaxy [7]. However, several Authors have proposed the use of various PCNL sheaths or nephroscopes equipped with suctioning mechanisms with promising outcomes [8–11]. Among the new instruments, the vacuum-assisted mini PCNL (vmPCNL) is a safe and effective treatment option for kidney stones [12, 13]. Previous Authors have shown that vmPCNL was associated with shorter OT, reduced use of accessory devices for stone removal and lower intra-pelvic pressures than mini-PCNL, thanks to its continuous aspiration system that allows for simultaneous lithotripsy and litholapaxy [13, 14]. Consequently, the rate of infectious complications was lower for vmPCNL than classic PCNL [13, 14]. Nonetheless, one of the major drawbacks of this system is its disposability, which may limit its use in the everyday clinical practice due to the fear of increasing the procedure-related costs.

We aimed to investigate clinical outcomes of patients with renal stones treated with vmPCNL or minimally invasive PCNL [11] at a single academic center and to perform a cost analysis between vmPCNL and MIP to explore potential predictors of costs associated with PCNL.

## Patients and methods

### Study cohort

We retrospectively analyzed all consecutive patients who underwent mini-PCNL for kidney stones at our tertiary-referral academic center between January 2016 and December 2020. All procedures from 01/2016 to 08/2017 were performed with the Minimally Invasive PCNL Set (MIP) (Karl Storz & Co. KG, Tuttlingen, Germany). Conversely, from 09/2017 to 12/2020 all mini-PCNL were carried out using the ClearPetra Set (Well Lead Medical Co., Ltd., China).

Patient’s demographics and medical history were collected. Body mass index (BMI) (kg/m^2^) was calculated for every patient. Comorbidities were scored with the Charlson comorbidity index (CCI) [15]. For the specific purpose of this study, the CCI was categorized as 0 vs. ≥ 1. A preoperative contrast enhanced computed tomography (CT) scan was requested. The stone volume was calculated using the ellipsoid formula (length × width × height × π × 1/6) [16]. Preoperative bladder urine culture was required for each patient. Patients with negative bladder urine culture were treated with one-shot II generation cephalosporin before surgery (if not contraindicated) [17]. Patients with asymptomatic bacteriuria started a targeted therapy 48–72 h before PCNL. In cases of leukocytosis, urinary symptoms or fever, the surgery was postponed after a full antibiotic course and a subsequent negative bladder urine culture.

All procedures were performed by two experienced (> 150 PCNL performed) endourologists (E.M; F.L.) and the surgical technique was standardized for both surgeons [14].

### Surgical technique

With the patient under general anesthesia, and placed in the supine Valdivia position, the procedure started with the placement of a ureteral catheter in the renal pelvis to inject contrast medium. Renal puncture was performed under combined fluoroscopic/ultrasonographic control and tract dilation was executed one-shot with the MIP 16 Ch metallic dilator, or with the ClearPetra sheath assembled with its stylet. A 550 μm Holmium: YAG laser fiber was used for stone fragmentation. Litholopaxy was performed by using the “vacuum-cleaner effect” during MIP, or through the aspiration-assisted sheath during vmPCNL. Flexible ureteroscope (7.9 Fr, Olympus URF-P6, Germany) and nitinol baskets were used through the percutaneous access when residual fragments could not be removed with the previous devices. An 8 Ch nephrostomy tube was positioned as exit strategy in all cases, while the ureteral catheter was left in place at the end of the procedure only after non-stone-free or complicated procedures, based on the surgeon’s preference.

### Intraoperative and postoperative data

The litholapaxy modality and OT (defined as the time from ureteral catheter placement to exit strategy) were recorded. According to internal protocol of our institution, uncomplicated procedures were managed as follows: on postoperative day one the bladder catheter was removed and the nephrostomy tube was closed; on postoperative day two a percutaneous pyelography was performed to assess ureteral canalization and the presence of residual stones. If ureteral canalization was confirmed, the nephrostomy tube was removed. Patients were discharged on postoperative day three.

Postoperative complications were graded according to the PCNL-adjusted Clavien Score [18]. Blood cultures were collected in case of fever (body temperature ≥ 38 °C) and/or chills after surgery. Postoperative antibiotic treatment was decided after consultation with the Infectious disease department.

Patients were evaluated within 3 months after surgery with abdominal ultrasound (US) or CT scan to identify residual stones. The stone free (SF) status was defined as the absence of residual fragments > 4 mm in diameter.

### Cost analysis

The accounts department of the hospital provided detailed expense reports, which were used to compare hospital costs for each procedure. We recorded the cost of surgical instruments, operation facilities, medications, laboratory services, radiology tests and any additional procedure that was performed for surgery-related complications (e.g. CT scan, angioembolization, placement of ureteral catheter). The operating fee was calculated using the operative time per the cost of the operative room equipe (two surgeons, one anesthesiologist, two nurses, one radiographer). The total procedural costs were calculated as the sum of the costs for the employment of the operative room (operating fee) and surgical instruments. The total hospitalization costs were calculated as the sum of the costs of overall hospitalization (procedural costs, laboratory, radiology, pharmacy, room and board) thus, including complication-related costs with any additional procedures, including readmission due to major complication and related procedures. Costs of instruments did not change in the study period. Common costs, including preoperative and postoperative visit costs, were not included in this study as routine management of PCNL patients.

A cohort of 225 patients who underwent miniPCNL (including vmPCNL and MIP) between 01/2016 and 12/2020 was identified. We excluded patients with congenital renal anomalies (*N* = 11); scheduled staged procedures for large stone burden (*N* = 30); concomitant additional procedures (*N* = 12); endoscopic combined intrarenal surgery (ECIRS) procedures (*N* = 2); stone fragmentation performed with ballistic, ultrasound or combined modality (*N* = 22); some patients presented more than one exclusion criteria. A sample of 120 (65.9%) and 62 (34.1%) patients treated with vmPCNL and MIP with complete perioperative and follow-up data was considered for statistical analyses.

Data collection followed the principles outlined in the Declaration of Helsinki. All patients signed an informed consent agreeing to share their own anonymous information for future studies. The study was approved by the Foundation IRCCS Ca’ Granda—Ospedale Maggiore Policlinico Ethical Committee (Prot. 25508).

### Statistical methods

We performed 1:2 propensity-score matching (nearest-neighbor analyses using a caliper width of 0.2 of the standard deviation of the logit of the propensity score) to control for measurable baseline differences among patients in the two groups [19]. Propensity scores were computed by modeling logistic regression with the dependent variable as the odds of being in the MIP group and the independent variables as age, BMI, CCI, stone volume, and stone location. After matching, 108 (66.7%) and 54 (33.3%) individuals in the vmPCNL and MIP group, respectively, were considered for the final analysis.

Distribution of data was tested with the Shapiro–Wilk test. Data are presented as medians (interquartile range; IQR) or frequencies (proportions). After matching, descriptive statistics were used to assess potential differences in terms of clinical parameters, intraoperative and postoperative characteristics between the MIP and the vmPCNL group. The statistical significance of differences in medians and proportions were tested with the Mann–Whitney *U* test and Fisher’s exact test, as indicated.

Univariable and multivariable linear regression models tested the association between clinical variables and total hospitalization costs in the whole cohort. Statistical analyses were performed using SPSS v.26 (IBM Corp., Armonk, NY, USA). All tests were two sided, and statistical significance level was determined at *p* < 0.05.

## Results

Before matching, stone volume [median (IQR) 2.6 (1.8–3.0) vs. 1.7 (1.2–3.4) cm^3^; *p* = 0.01] was higher in the MIP than the vmPCNL group. There was also a significant difference in stone location between groups before matching (*p* = 0.03). After matching, patients and perioperative characteristics were evenly distributed.

Table 1 details descriptive statistics of the whole cohort according to the type of surgery after matching. OT was shorter during vmPCNL than MIP procedures [89 (73–126) vs. 115 (90–160) min; *p* < 0.001]. The use of flexible ureteroscopes and baskets to complete litholapaxy was more frequently reported during MIP than vmPCNL (all *p* ≤ 0.01). Hospital stay was longer after MIP than vmPCNL procedures (*p* = 0.03) and the SF rate was higher after vmPCNL than MIP (*p* = 0.04).

**Table 1:** Demographic characteristics and descriptive statistics of patients according to the type of surgery after matching

	vmPCNL	MIP	*p* value*
No. of individuals	108 (66.7%)	54 (33.3%)	
Age (years)			0.7
Median (IQR)	56 (50–75)	56 (50–76)	
Range	22–81	23–82	
Male Gender [No. (%)]	65 (60.2)	37 (68.5)	0.5
BMI (kg/m^2^)			0.7
Median (IQR)	25 (23–27)	25 (22–28)	
Range	19–41	19–40	
CCI ≥ 1 [No. (%)]	31 (28.7)	17 (31.4)	0.6
Laterality [No. (%)]			0.8
Right	48 (44.5)	23 (42.6)	
Left	60 (55.5)	31 (57.4)	
Stone volume (cm^3^)			0.5
Median (IQR)	2.2 (1.1–3.7)	2.2 (1.1–3.5)	
Range	0.6–19.0	0.5–19.0	
Single stone [No. (%)]	43 (39.8)	22 (40.7)	0.8
Stone location			0.3
Upper pole calices	17 (15.7)	9 (16.6)	
Mid pole calices	38 (35.1)	20 (37.1)	
Lower pole calices	69 (63.8)	32 (59.3)	
Pelvis	43 (39.8)	25 (46.2)	
Staghorn stone [No. (%)]	34 (31.5)	17 (31.4)	0.6
Stone density (Hounsfield unit)			0.5
Median (IQR)	1034 (891–1501)	1041 (743–1444)	
Range	176–2290	460–2032	
Operative time (min)			< 0.001
Median (IQR)	89 (73–126)	115 (90–160)	
Range	35–210	60–255	
Litholapaxy with basket [No. (%)]	42 (38.8)	50 (92.6)	< 0.001
Use of flexible ureteroscope [No. (%)]	44 (40.7)	33 (61.1)	< 0.01
Exit strategy [No. (%)]			0.1
Nephrostomy only	83 (76.8)	35 (64.8)	
Nephrostomy + Ureteral catheter	25 (23.2)	19 (35.2)	
Hospitalization time (days)			0.03
Median (IQR)	4 (3–5)	5 (3–6)	
Mean (SD)	4.2 (3.1)	5.8 (3.7)	
Range	2–12	2–14	
Hemoglobin drop (g/dL)			0.2
Median (IQR)	− 1.5 (− 1.9 to − 0.9)	− 1.6 (− 2.8 to − 0.7)	
Range	− 5.1 to − 0.1	− 6.0 to − 0.2	
Stone free rate [No. (%)]	98 (90.7)	42 (79.6)	0.04
Auxiliary procedures [No. (%)]			0.08
No treatment	6 (5.5)	7 (12.9)	
RIRS	2 (1.9)	2 (3.7)	
Second look PCNL	2 (1.9)	3 (5.5)	

*vmPCNL* vacuum-assisted miniPCNL, *MIP* minimally invasive PCNL, *BMI* body mass index, *CCI* Charlson Comorbidity Index, *PCNL* percutaneous nephrolithotomy, *RIRS* retrograde intrarenal surgery;

*p value according to the Mann–Whitney *U* test and Fisher’s exact test, as indicated

The rate of infectious complications was higher after MIP than vmPCNL (24.0% vs. 8.3%, *p* < 0.01) (Table 2). Conversely, rates of overall postoperative complications were similar between groups (38.5% for the MIP vs. 24.1% for vmPCNL; *p* = 0.07). Clavien-Dindo grade > II complications were found in 7 (6.4%) and 4 (7.4%) patients after vmPCNL and MIP (*p* = 0.3), respectively (Table 2).

**Table 2:** Postoperative complications in the whole cohort after matching (No. = 162)

	vmPCNL (*N* = 108)	MIP (*N* = 54)	*p* value*
Overall complications [No. (%)]	26 (24.1)	21 (38.8)	0.07
Highest Clavien-Dindo [No. (%)]			0.3
I–II	19 (17.6)	17 (31.4)	
IIIa–IIIb	7 (6.4)	4 (7.4)	
Blood transfusion [No. (%)]	1 (0.9%)	2 (3.7%)	0.2
Infectious complications			
Any Clavien-Dindo [No. (%)]	9 (8.3)	13 (24.1)	< 0.01
Readmission [No. (%)]	4 (3.7)	2 (3.7)	0.9
Hemothorax	1	0	
Urine leakage	1	0	
Hematuria	2	2	

*vmPCNL* vacuum-assisted miniPCNL, *MIP* Minimally invasive PCNL, *PCNL* Percutaneous nephrolithotomy

*p value according to the Fisher Exact test

Table 3 reports the basic equipment for each procedure and related costs. The overall cost for the surgical instruments of PCNL procedure was higher for vmPCNL than MIP (*p* < 0.001). Supplementary Table 1 depicts costs associated with additional equipment, laboratory or radiologic tests and procedures.

**Table 3:** Basic equipment and related cost for PCNL procedures

Instrument	*N*	Cost for one unit (Euros)	Cost for one procedure (Euros)
General PCNL procedure			
Sterile gynecological drape	1	22.57	22.57
Nephroscopy surgical drape	1	10	10
Medical camera drape	2	1.07	2.14
Laser sterile drape	1	0.8601	0.8601
C-arm surgical drape	1	35.883	35.883
Sterile surgical gowns	5	3.6	18
Sterile surgical glove	5	0.7564	3.782
2-ways irrigation set	1	1.1956	1.1956
Sterile suction tube	1	1.444	1.444
Sterile fluid warming irrigation set	1 (cost calculated for 4 uses)	107.36	26.84
Sterile patient line irrigation set	1	25.62	25.62
20 ml syringe	6	0.0488	0.2928
60 ml syringe	2	0.1326	0.2652
Foley bladder catheter ch 16	1	1.2078	1.2078
Antiseptic applicator 10.5 ml	2	3.41	6.82
Sodium chloride 0.9% 2000 ml	2	1.2031	2.4062
Sterile urine bag	2	1.464	2.928
1% lidocaine gel	2	1.892	3.784
Hydrophilic guidewire	2	24.278	48.556
Ultrasound probe drape	1	5.49	5.49
Connector adapter	1	5.002	5.002
Ureteral catheter ch 6	1	10.004	10.004
8ch percutaneous nephrostomy set	1	73.2	73.2
0 silk suture	2	0.7137	1.4274
Iopamidol 300–200 ml	1	23.76	23.76
Laser fiber 550 µm	1 (cost calculated for 10 uses)	888	88.80
MIP			
Nephrostomy tract dilators	1 × 72 procedures (yearly)	195.58	5.42
Nephrostomy sheath	1 × 72 procedures (yearly)	542.08	15.06
		Total for MIP	441.99
vmPCNL			
16 Ch nephrostomy sheath	1	256.2	256.2
Stone collection bottle	1	10.12	10.12
		Total cost for vmPCNL	687.82

*PCNL* Percutaneous nephrolithotomy, *vmPCNL* vacuum-assisted miniPCNL, *MIP* Minimally invasive PCNL

The cost-analysis of vmPCNL and MIP is shown in Table 4. Total procedural costs were equivalent between groups [999.7 (922.1–1158.4) € for MIP and 1000.7 (924.7–1161.3) € for vmPCNL, *p* = 0.9]. Conversely, costs related to antibiotics (*p* < 0.01) and additional radiological/laboratory examinations for the postoperative management of complications were higher for MIP than vmPCNL (all *p* ≤ 0.02). The cost for in-hospital complications was higher for MIP than vmPCNL [mean (SD) 89.3 (50.3) € vs. 34.8 (27.6) €; *p* < 0.01]; similarly, the overall cost for the hospitalization was higher for MIP than vmPCNL [2658.2 (2084.4–3143.1) € vs. 2302.9 (1976.1–2693.1) €; *p* = 0.01]. After discharge, 2 (3.7%) and 4 (3.7%) patients in the MIP and vmPCNL group, respectively, were re-admitted due to PCNL-related complications (*p* = 0.9). Specifically, 2 patients in the MIP group were readmitted for postoperative hematuria; conversely, hemothorax (*N* = 1), urine leakage (*N* = 1) and hematuria (*N* = 2) were causes for readmission after vmPCNL (Table 2). Costs associated with hospital readmission were similar between groups (Table 4).

**Table 4:** Cost analysis between vmPCNL and MIP (No. = 162)

Cost (Euros)	vmPCNL	MIP	*p* value*
	(*N* = 108)	(*N* = 54)	
Operating fee			< 0.001
Median (IQR)	253.8 (211.5–352.5)	338.4 (310.2–447.6)	
Range	101.5–592	169.2–724.7	
Additional surgical equipment			
Basket			< 0.001
Median (range)	62.2 (0–162.5)	162.5 (0–325)	
Nephrostomy tube			0.2
Mean (range)	6.5 (0–6.7)	12.1 (0–67)	
Total procedural costs			0.9
Median (IQR)	1000.7 (924.7–1161.3)	999.7 (922.1–1158.4)	
Range	790.1–1501.4	679–1541.8	
Post-operative costs			
Antibiotic			< 0.01
Median (IQR)	3.3 (3.2–13.1)	8.2 (3.8–27.5)	
Range	1.1–491.2	1.6–316.4	
Radiology test			< 0.01
Mean (SD)	6.7 (4.3)	24.9 (15.7)	
Range	0.0–199	0.0–455.4	
Blood culture			0.02
Mean (range)	0.7 (0–19)	1.9 (0–19)	
Transfusion			0.1
Mean (range)	1.96 (0–200)	44.3 (0–1350)	
Additional procedures			0.4
Mean (range)	18.4 (0–542.9)	25.4 (0–2370.9)	
In hospital complications			< 0.01
Mean (SD)	34.8 (27.6)	89.3 (50.3)	
Range	0.0–2394.9	0.0–1805.4	
Hospital stay			< 0.01
Median (IQR)	1200 (900–1500)	1500 (1200–1800)	
Range	600–3600	600–4200	
Total hospitalization cost			0.01
Median (IQR)	2302.9 (1976.1–2693.1)	2658.2 (2084.4–3143.1)	
Range	1546.4–7225.9	1631.3–6235.9	
Complications after discharge			0.8
Mean (SD)	204.3 (129.5)	172.8 (152.5)	
Range	0.0–8942	0.0–5327.1	

*vmPCNL* vacuum-assisted miniPCNL, *MIP* Minimally invasive PCNL, *PCNL* Percutaneous nephrolithotomy, *OR* Operative room

*p value according to the Mann–Whitney test

Table 5 depicts linear regression models testing the association between clinical variables and total costs of hospitalization. Univariable linear regression models revealed that CCI ≥ 1, OT, the presence of postoperative complications (any), and length of stay (LOS) were all significantly associated with total hospitalization costs (all *p* < 0.001). Conversely, vmPCNL was associated with a reduced hospitalization cost, compared to MIP (beta − 411.4€; 95% CI − 697.1€ to − 125.9€; *p* < 0.01). Multivariable linear regression analysis revealed that each day of hospitalization contributed 334.2 € (95% CI 302.4€–365.9€; *p* < 0.001) to the overall cost model, while for one hour increase in OT, the total hospitalization cost will increase by 234€ (95% CI 144€–330€, *p* < 0.001). Postoperative complications, CCI and the type of surgery were adjusted for in the cost model but did not affect total hospitalization costs.

**Table 5:** Linear regression models predicting total costs of hospitalization in the whole cohort after matching

	UVA model			MVA model		
	Beta	*p* value	95% CI	Beta	*p* value	95% CI
Age	4.1	0.5	− 6.2 to 14.5			
CCI ≥ 1	297.3	0.04	7.9–586.7	106.6	0.09	− 17.1 to 230.6
BMI	13.1	0.4	− 19.6 to 45.6			
Stone volume	20.5	0.6	− 27.9 to 68.8			
Operative time (min)	8.2	< 0.001	5.1–11.3	3.9	< 0.001	2.4–5.5
Postoperative complications	747.6	< 0.001	488.7–1006.4	55.1	0.4	− 80.1 to 190.4
Hospitalization time (days)	357.1	< 0.001	327.5–386.6	334.2	< 0.001	302.4–365.9
vmPCNL vs. MIP	− 411.4	< 0.01	− 697.1 to − 125.9	− 215.4	0.5	− 413.8 to 10.2

*UVA* univariate model, *MVA* multivariate model, *CCI* Charlson Comorbidity Index, *BMI* body mass index, *MIP* minimally invasive PCNL, *CI* confidence interval

**Fig. 1:**
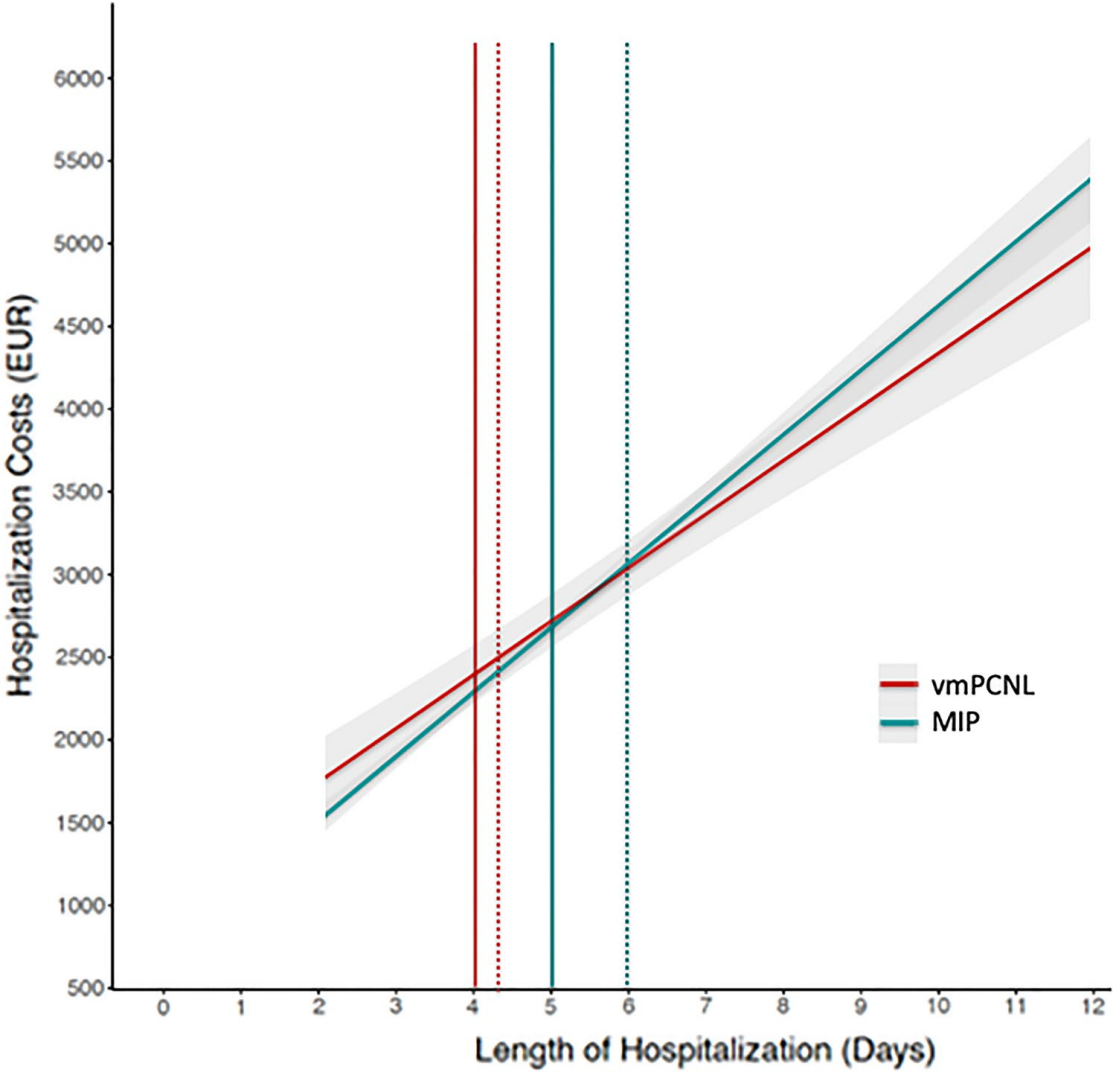
Smoothed linear regression analysis of length of stay versus hospitalization cost stratified by vacuum assisted PCNL (vmPCNL) (red) and minimally invasive PCNL (MIP) (green). Gray areas represent 95% confidence intervals. Vertical continuous and dashed lines display median and mean length of stay for each surgical technique, respectively. *PCNL* Percutaneous nephrolithotomy

**Fig. 2:**
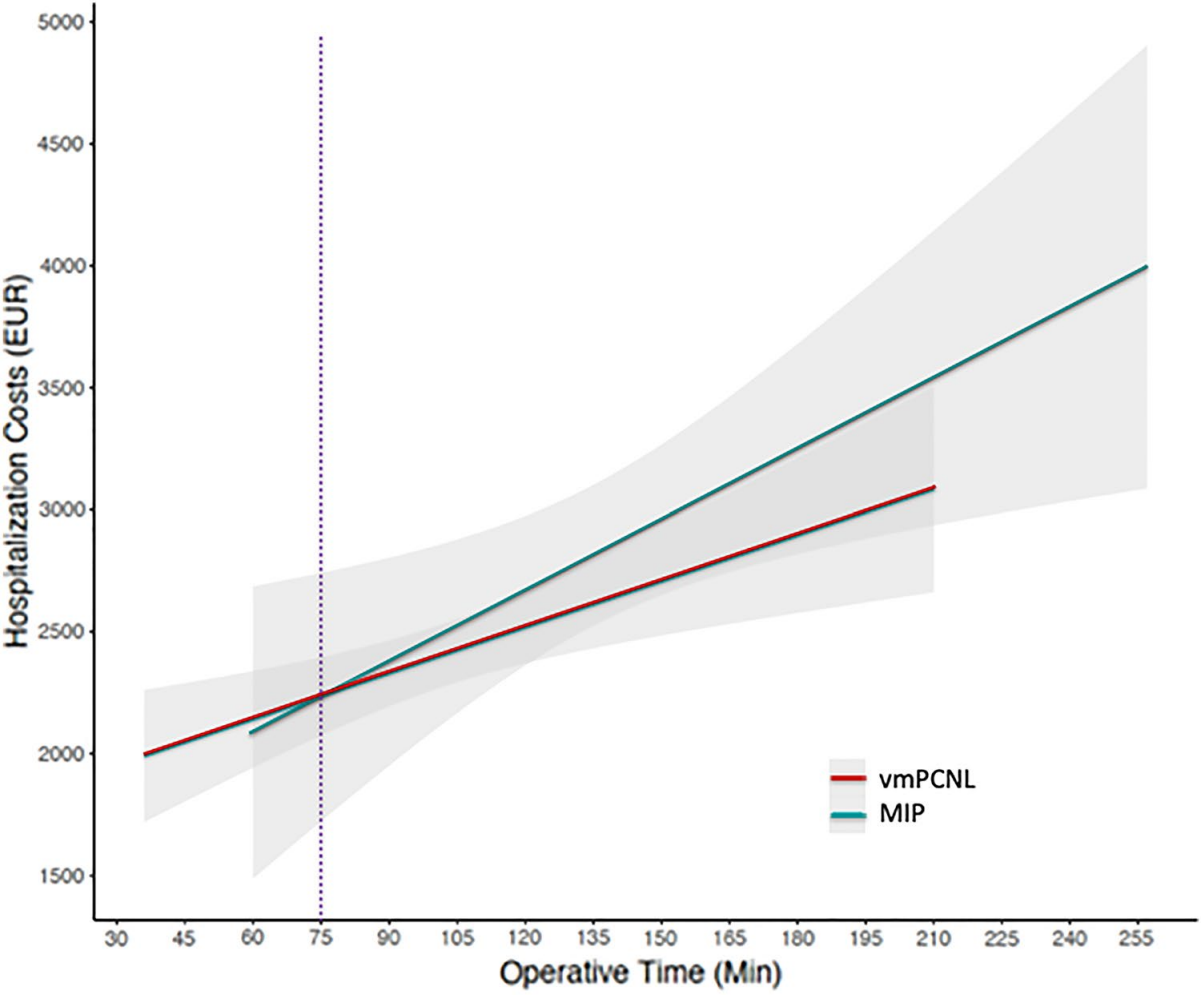
Smoothed linear regression analysis of operative time versus hospitalization cost stratified by vacuum assisted PCNL (vmPCNL) (red) and minimally invasive PCNL (MIP) (green). Gray areas represent 95% confidence intervals. Vertical dashed line displays the point of cost equivalence between the two groups. *PCNL* Percutaneous nephrolithotomy

The cost model suggested that a savings of approximately 1 day of hospitalization is required to offset the costs associated with the use of vmPCNL, as shown in the linear regression plots comparing hospitalization cost by LOS, stratified by surgical approach (Fig. 1). The point of cost equivalence for a vmPCNL case staying approximately 4 days is between 5 and 6 days with the MIP approach. Similarly, Fig. 2 represents the linear regression model showing the association between OT and hospitalization cost stratified by the two surgical techniques. The point of cost equivalence between vmPCNL and MIP was approximately 75 min, after which vmPCNL was less costly than MIP procedures (Fig. 2).

## Discussion

In this cross sectional study, we compared costs associated with vmPCNL and MIP procedures for kidney stones. We showed that, despite the additional cost of the disposable nephrostomic sheath, total procedural costs were comparable between groups, but total hospitalization costs were lower for vmPCNL than MIP.

The PCNL technique has evolved in the last decades, and one of the most recent advancement is the aspiration-assisted system, first introduced by Zeng et al. [20, 21]. High stone free rates, low complication rates, fast stone disintegration and shorter OT were all important characteristics of suction PCNL procedure [22]. vmPCNL, in particular, was associated with shorter OT, lower rates of infectious complications and lower need for additional equipment than classic mini-PCNL [14]. However, there is a lack of reports that specifically investigated costs associated with suction assisted PCNL in the real-life setting.

Our study showed that, despite the overall cost for the surgical instruments of PCNL was higher for vmPCNL than MIP, the total procedural costs were comparable between groups. Indeed, vmPCNL was associated with shorter OT than MIP, which was the main factor determining the operating fee. Moreover, shorter OT may help limiting infectious complication by reducing the time span during which surgery is performed at elevated intra-pelvic pressure. Aspiration-assisted mPCNL techniques have already been proved to be associated with less bleeding [10, 23], lower renal pelvic pressures and subsequent infectious complications [6, 12, 14, 24] than non-suctioning miniPCNL procedures. In this series we confirmed that infectious complications were lower for vmPCNL than MIP. Besides clinical implications, this finding had a relevant impact over the total hospitalization costs, thanks to the reduction of the expenses related to post-operative antibiotic therapies, radiological and laboratory tests and complication-related ancillary procedures. Infectious complications were associated with both greater hospitalization cost and increased LOS, supporting that cost savings with vmPCNL may be directly tied to expediting convalescence and reduced morbidity.

Similar to previous reports [23, 25] we observed a shorter LOS in the vmPCNL group than the MIP group. Consequently, the costs related to the hospital stay were significantly lower for vmPCNL and contributed to the lower total hospitalization costs of the vmPCNL compared to MIP.

The importance of this study is severalfold. First, it is the first report of a detailed cost analysis comparison between vmPCNL and MIP procedures. Second, we showed that vmPCNL was associated with a significantly shorter hospital stay, shorter OT and lower rate of perioperative complications compared to MIP, suggesting that expedited convalescence can compensate for the expenses associated with the disposable sheath. As reported by our linear model, by means of vmPCNL surgery approximately 1 day of hospitalization needs to be saved to make up for the costs associated with the disposable set. When examining cases where LOS was 4 days or less (the median hospitalization time for vmPCNL in our cohort), MIP was cheaper suggesting that cost equivalence is being primarily driven by lower perioperative morbidity and subsequent shorter LOS for vmPCNL. This was further confirmed by the multivariable model where the effect of LOS and OT were independent from other factors known to influence hospitalization cost, thus including vmPCNL procedures.

In terms of OT the linear regression model suggests that, for procedure lasting more than 75 min, vmPCNL is more cost-convenient than MIP. Adding this to the demonstrated clinical benefit of vmPCNL, in terms of lower rates of infectious complications and shorter hospital stay, it appears reasonable to privilege vmPCNL in cases in which a longer procedure is foreseen for stone’s or patient’s characteristics. However, despite being novel and innovative, these findings deserve validation from randomized clinical trials comparing aspiration and non-aspiration assisted mini-PCNL techniques.

Collectively, these findings imply that vmPCNL is potentially a cost equivalent option as MIP with faster convalescence and minimal peri-operative morbidity in appropriately selected patients.

This study is not devoid of limitations. First, although propensity-score matching analysis is a solid method to reduce the selection bias of a retrospective study, the lack of randomization may limit the interpretation of our results. Second, this was a single center-based study, which raises the possibility of further selection biases Therefore, larger studies across different centers and cohorts are needed to externally validate our findings. Moreover, due to our internal protocol, median LOS was > 3 days in this cohort, meanwhile hospitalization for uncomplicated PCNL is progressively shortening, especially in high volume centers [26]. Given the optimal results with these new technologies, we foresee a reduction of LOS, which may further confirm the feasibility of using single use surgical equipment. In addition, cost assessment might vary by hospital, in particular for high capital cost expenditures such as the disposable access sheath, which can be variably accounted for and in which cost-per-use will likely be lower at higher volume institutions. While this may limit applicability to any individual hospital/practice, we believe that our novel results should drive the organization of a population-based dataset that would be more representative of national outcomes compared to data from individual high-volume surgical centers.

## Conclusions

While miniaturized PCNL remains the preferred treatment option for kidney stones, our findings suggest vmPCNL may represent an attractive option due to clinical and economic benefits. The shorter OT and the lower need for disposable equipment during vmPCNL surgery reduced the procedural costs and offset the use of the disposable set. Moreover, vmPCNL was characterized by lower total hospitalization costs due to the lower rate of infectious complications and associated costs (antibiotic therapies, radiology and laboratory tests, longer hospitalization). Future larger studies are needed to explore the true cost benefit of vmPCNL over classic miniaturized PNCL.

## Supplementary Information

Below is the link to the electronic supplementary material.

Supplementary file1 (DOC 57 KB)

## Data Availability

None.
