# Supplementary material for: Cost analysis between mini-percutaneous nephrolithotomy with and without vacuum-assisted access sheath
Source: World J Urol. 2021 Aug 25;40(1):201–11. doi: 10.1007/s00345-021-03811-5 (PMC8813798; doi:10.1007/s00345-021-03811-5)
Supplement: Supplementary file 1 — Supplementary file1 (DOC 57 KB) [file 345_2021_3811_MOESM1_ESM.doc]

**Supplementary Table 1. Cost for additional equipment, labs and procedures**

| **Additional equipment** | Cost for one unit (Euros) |
| --- | --- |
| DJ stent | 54.90 |
| Basket | 162.56 |
| **Radiological procedures** |  |
| Abdominal ultrasound | 13 |
| Abdominal/chest X-Ray | 7.55 |
| Non-CE Abdominal CT | 24.45 |
| CE Abdominal CT | 76.60 |
| Pyelography | 27.90 |
| NCE head CT | 21.20 |
| PICC line | 192.23 |
| **Laboratory testing** |  |
| Blood cultures (negative) | 7 |
| Blood cultures (positive) | 19 |
| **Transfusion** |  |
| Blood | 100 |
| Platelets | 150 |
| **Antibiotic** |  |
| Amikacin 500 mg EV | 0.59 |
| Amoxicillin/Clavulanic acid 875/125 mg OS | 0.12 |
| Ampicillin 1000 mg EV | 3.99 |
| Ampicillin/Sulbactam 3000 mg EV | 11.98 |
| Aztreonam 2000 mg EV | 8.80 |
| Trimethoprim- sulfamethoxazole  1000 mg EV | 2.17 |
| Trimethoprim- sulfamethoxazole  450 mg EV | 1.09 |
| Trimethoprim- sulfamethoxazole  1000 mg OS | 0.82 |
| Cefazolin 1000 mg EV | 1.65 |
| Cefixime 400 mg OS | 0.74 |
| Cefotaxime 2000 mg EV | 1.69 |
| Cefoxitin 1000 mg EV | 3.28 |
| Ceftazidime 2000 mg EV | 4.35 |
| Ceftriaxone 1000 mg EV | 0.63 |
| Ciprofloxacin 400 mg EV | 1.14 |
| Ciprofloxacin 500 mg OS | 0.07 |
| Clindamycin 600 mg EV | 0.76 |
| Daptomycin 750 mg EV | 54.07 |
| Fluconazole 100 mg OS | 0.11 |
| Fosfomycin 800 mg EV | 60.96 |
| Gentamicin 80 mg EV | 0.46 |
| Imipenem/Cilastatin 500+500 mg | 2.40 |
| Metronidazole 500 mg EV | 0.28 |
| Imipenem 500 mg EV | 2.89 |
| Meropenem 500 mg EV | 1.26 |
| Nitrofurantoin 100 mg | 0.06 |
| Piperacillin/Tazobactam 4500 mg EV | 2.34 |
| Vancomycin 500 mg EV | 1.45 |

Costs are calculated on the average execution time of the exam, the hourly cost of the staff involved, and the cost of the disposable used in each procedure.

Diagnostics and therapeutics angiography costs were based on the average execution time of the procedure, the hourly cost of the staff involved, and the cost of the materials used in each individual procedure

Keys: DJ = Double J stent; NCE = Non contrast enhanced; CT = computerized tomography; CE= contrast enhanced; PICC= peripherally inserted central catheter; EV= endovenous
